# HIPK2 in Angiogenesis: A Promising Biomarker in Cancer Progression and in Angiogenic Diseases

**DOI:** 10.3390/cancers15051566

**Published:** 2023-03-02

**Authors:** Alessia Garufi, Valerio D’Orazi, Giuseppa Pistritto, Mara Cirone, Gabriella D’Orazi

**Affiliations:** 1 Unit of Cellular Networks, Department of Research and Advanced Technologies, IRCCS Regina Elena National Cancer Institute, 00144 Rome, Italy; 2 Department of Surgery, Sapienza University, 00185 Rome, Italy; 3 Centralized Procedures Office, Italian Medicines Agency (AIFA), 00187 Rome, Italy; 4 Laboratory Affiliated to Pasteur Institute Italy Foundation Cenci Bolognetti, Department of Experimental Medicine, Sapienza University of Rome, 00161 Rome, Italy; 5 Department of Neurosciences, Imaging and Clinical Sciences, University “G. D’Annunzio”, 66013 Chieti, Italy

**Keywords:** VEGF, HIF-1, hypoxia, micro-RNA, circular HIPK2, p53, cancer, diabetes, diabetic retinopathy, wound healing

## Abstract

**Simple Summary:**

Dysregulated angiogenesis contributes to cancer progression and to many chronic inflammatory diseases. Many efforts in the field of angiogenesis have been made to discover new potential molecular targets to be used as biomarkers or to improve the anti-angiogenic therapies. HIPK2, an oncosuppressor able to regulate multiple molecular pathways, has been shown lately to play a role in angiogenesis both in cancer and in other angiogenic diseases. Therefore, HIPK2 emerges as a potential new biomarker of angiogenic diseases.

**Abstract:**

Angiogenesis is the formation of new blood capillaries taking place from preexisting functional vessels, a process that allows cells to cope with shortage of nutrients and low oxygen availability. Angiogenesis may be activated in several pathological diseases, from tumor growth and metastases formation to ischemic and inflammatory diseases. New insights into the mechanisms that regulate angiogenesis have been discovered in the last years, leading to the discovery of new therapeutic opportunities. However, in the case of cancer, their success may be limited by the occurrence of drug resistance, meaning that the road to optimize such treatments is still long. Homeodomain-interacting protein kinase 2 (HIPK2), a multifaceted protein that regulates different molecular pathways, is involved in the negative regulation of cancer growth, and may be considered a “bona fide” oncosuppressor molecule. In this review, we will discuss the emerging link between HIPK2 and angiogenesis and how the control of angiogenesis by HIPK2 impinges in the pathogenesis of several diseases, including cancer.

## 1. Introduction

Angiogenesis is the formation of new blood capillaries taking place from preexisting functional vessels. In the adult, a physiologic vessels formation is transiently activated for tissue growth and regeneration during processes such as wound healing and the female reproductive cycle. However, angiogenesis may also have a pathologic role as it fuels inflammatory and malignant diseases [1]. Deregulation of the normal vessels’ growth is observed in many diseases including diabetic retinopathy, autoimmune diseases, rheumatoid arthritis, atherosclerosis, cerebral ischemia, cardiovascular diseases, psoriasis, and delayed wound healing [2]. In many solid cancers, angiogenesis is constantly activated by the “angiogenic switch” that causes normally quiescent vasculature to continually sprouts new vessels. In this way, angiogenesis helps to sustain expanding neoplastic growth. For that reason, angiogenesis is considered a hallmark of cancer progression [3,4]. The interaction between neoplastic cells by means of the angiogenic factors produced by them, and the newly formed vessels promotes the growth of solid tumors and the metastases formation, as well as the impairment of the efficacy of anticancer therapies [5]. Interestingly, other than the tumor cells, there are also stromal cells such as tumor-associated macrophages (TAM) which can produce the angiogenic factors that promote angiogenesis and metastasis [6].

Many reviews have extensively summarized the steps through which the vascular bed expands by sprouting and matures into a system of stable vessels in normal and pathological conditions; therefore, here the main molecules regulating angiogenesis will be only briefly described. Angiogenesis is regulated by the balance of many positive and negative factors released into the microenvironment [7,8]. The positive regulators include vascular endothelial growth factors (VEGFs); A, B, and C fibroblasts growth factors (FGFs); 1 and 2 platelet-derived growth factor (PDGF); hepatocyte growth factor (HGF); and angiopoietins, while the negative regulators include angiostatin, endostatin, thrombospondin, and interferons [8]. However, the most important mediator of angiogenesis is VEGFA, which acts on endothelial cells by binding two different receptors (R), namely VEGFR-1 and VEGFR-2 [9]. The binding of VEGF to its receptor activates the PI3K/Akt, MEK, or FAK signaling pathways leading to the expression of genes whose proteins induce vascular permeability, cell proliferation, and motility, thus promoting angiogenesis [10]. Since its discovery, VEGF has revolutionized the comprehension of the angiogenesis process in normal tissue development and in health conditions, as well as in the course of many diseases [11]. The targeting of VEGF is therefore a therapeutic approach of high interest and, so far, hundreds of thousands of patients have been treated with blockers of VEGF even if the limited therapeutic efficacy due to the activation of resistance mechanisms remains an outstanding problem [8,12].

A key driver of angiogenesis is the hypoxia-inducible factor-1 (HIF-1), a heterodimeric transcription factor that consists of two subunits: the oxygen-sensitive subunit HIF-1α (or its analogs HIF-2α and HIF-3α) that undergoes quick degradation under normoxic conditions and the constitutively expressed HIF-1β, also known as aryl hydrocarbon nuclear translocator (ARNT) [13,14]. Under hypoxia, HIF-1α is stabilized via several post-translational modifications involving hydroxylation, acetylation, and phosphorylation. Following activation, HIF-1α translocates into the nucleus to bind HIF-1β and induce the transcription of several target genes involved in many aspects of cancer progression including angiogenesis (e.g., VEGF, PDGFB), metabolic adaptation (e.g., GLUT1, PDK1), apoptosis resistance (e.g., Bcl-2, MDR), invasion, and metastasis (e.g., CXCR4, MMP9) [15,16]. When a tumor mass grows beyond 1–2 mm, it undergoes hypoxia because of the distance from the host microvasculature, which makes it difficult to efficiently supply the tumor with nutrients and oxygen. In order to survive to the hypoxia, tumors activate HIF-1 [16]. Other than hypoxia, many genetic alterations inactivating tumor suppressors or activating oncoproteins have been reported to increase the basal levels of HIF-1α in cancers and contribute to tumor progression and angiogenesis [17].

## 2. HIPK2 and Tumor Angiogenesis

Homeodomain-interacting protein kinase-2 (HIPK2) is a serine/threonine kinase which belongs to a family that includes four members (HIPK1, HIPK2, HIPK3, and HIPK4) of corepressors for homeodomain transcription factors whose structures and functions have been extensively summarized (for a review, see references [18,19,20]). HIPK2 modulates the activity of many transcriptional regulators and chromatin modifiers and, depending on the cell context, it can repress or promote the gene transcription [21]. HIPK2 regulates the expression of several genes involved in cell development, cytokinesis, protein stability, apoptosis, and DNA damage response [22]. One of the most important targets of HIPK2 is the oncosuppressor p53 that is phosphorylated by HIPK2 in Serine 46 to specifically activate the p53 apoptotic function essential for the success of the anticancer therapies [23,24,25,26]. HIPK2 may also regulate p53-independent pathways and, for this reason, HIPK2 dysregulation is associated with neurological diseases and fibrosis other than with cancer progression [20,27]. Recently, a role for HIPK2 in angiogenesis has been pointed out, not only in cancer, but also in other angiogenic diseases, and will be summarized below.

### 2.1. HIPK2 and HIF-1/VEGF in Tumor Angiogenesis

**Figure 1:**
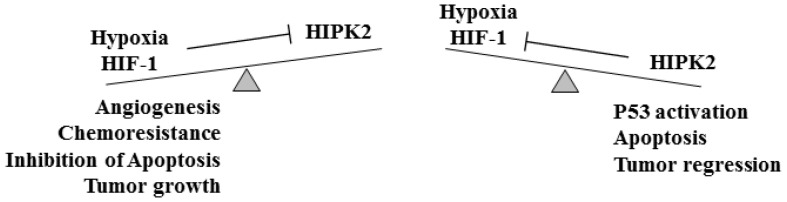
Schematic representation of the balance between hypoxia and HIPK2 in cancer. When hypoxia is activated ((**left**) panel) the hypoxia-inducible mechanisms inhibit HIPK2 and the effects of hypoxia (such as angiogenesis, chemoresistance, inhibition of apoptosis, and tumor growth) prevail. When HIPK2 is activated ((**right**) panel) the HIF-1-induced molecular mechanisms are inhibited and the antitumor effects (such as p53 activation, activation of apoptosis, and tumor regression) prevail.

Our previous studies showed that HIPK2 binds, along with histone deacetylase 1 (HDAC1), to the HIF-1α gene promoter repressing the HIF-1-mediated transcription of many target genes including VEGF, therefore restraining tumor growth [28]. As a proof of principle, HIPK2 silencing with small interfering RNA upregulated HIF-1α in cancer cells, inducing a pseudohypoxic tumor phenotype in normoxic conditions, fueling tumor progression, and chemoresistance [28]. To examine whether the effect of HIPK2 on the modulation of HIF-1α/VEGF pathway was associated with endothelial cell sprouting, the growth of human umbilical vein endothelial cells (HUVEC) was evaluated in vitro in the presence of conditioned media (CM) derived from colon cancer cells depleted or not of the HIPK2 function, and the results confirmed that HIPK2 silencing increases tumor vascularity in vitro [28]. Interestingly, hypoxia-driven mechanisms lead to HIPK2 protein degradation [29]. Therefore, a regulatory loop exists between HIPK2 and HIF-1α that affects the multiple downstream molecular pathways, including p53 and VEGF, regulated by both proteins [30,31,32], impinging on tumor growth and angiogenesis and/or on tumor regression (Figure 1).

Thus, HIPK2 silencing increases the xenograft tumor growth and the physiologic relevance was assessed by analyzing the HIPK2 gene expression in human specimens collected from patients with the familial adenomatous polyposis (FAP) and with sporadic colorectal cancer (CRC). HIPK2 mRNA levels were lower in sporadic CRC tissues compared to FAP tissues and the HIPK2 expression in human CRC inversely correlate with the staging of the tumors [33], although the molecular mechanisms leading to HIPK2 mRNA downregulation were not unveiled. In the attempt to target hypoxia and restrain tumor angiogenesis, we have shown that zinc chloride induces HIF-1α protein degradation and inhibits the HIF-1-induced transcription of VEGF and angiogenesis, in vitro and in animal studies [34]. In addition, zinc counteracts the hypoxia-induced HIPK2 deregulation restoring p53 apoptotic response to chemotherapy, underscoring the potential use of zinc supplementation in combination with chemotherapy to improve the efficacy of the anticancer treatments [35,36].

The balance between HIPK2 and HIF-1 in angiogenesis was recently confirmed in a study on hepatocellular carcinomas (HCC) [37]. In two independent patients’ cohorts with, respectively, 90 and 52 paired HCC and adjacent normal tissues, the authors analyzed using immunohistochemistry (IHC) of the expression levels of HIPK2 protein and found that they were lower in the cancer tissues compared to the adjacent normal tissues [37]. Studies performed in animal models showed that HIPK2 overexpression reduces tumor xenografts growth and metastasis formation. IHC analysis of the xenograft tumor tissues derived from Huh7 or BEL-7404 cancer cells, with or without HIPK2 overexpression, showed that HIPK2 downregulation significantly increases VEGFα level in the subcutaneous tumor and in the corresponding new blood vessels [37]. Following this, in vitro studies evaluated the tube formation of HUVEC cultured with the conditioned medium (CM) of HCC cells with or without HIPK2 overexpression in hypoxic condition. The tube formation was reduced when HUVEC were cultured with the medium derived from cells with HIPK2 overexpression compared with the medium derived from the control cells [37], strengthening the finding that HIPK2 may inhibit hypoxia-induced angiogenesis in the HCC tumor, as observed in the above reported study on CRC [28]. Mechanistically, the authors found that HIPK2 directly binds to and downregulates HIF-1α protein by inducing its proteasomal degradation as demonstrated by the use of the proteasome inhibitor MG132 [37]. To further evaluate the antiangiogenic role of HIPK2 in HCC samples, the authors analyzed data retrieved from the Gene Expression Omnibus (GEO) database. They found lower HIPK2 expression in both metastasis tissues and the primary lesions with metastasis compared to the primary lesions without metastasis. After this, by knocking out with the CRISPR-Cas9 system the HIF-1α and HIPK2 genes individually or simultaneously, the authors determined the in vivo tumor growth capacities of Hepa1-6 cells in a xenograft mice model. They observed enhanced in vivo tumor growth of the HIPK2−/− cells while the tumor cells with HIF-1α knockout grew significantly slower compared to the control tumors [37]. The double HIF-1α and HIPK2 knockout greatly counteracted the tumor growth caused by the HIPK2 knockout, suggesting that the effect of HIPK2 depletion on HCC progression was mediated by HIF-1α and by the HIF-1-induced angiogenesis [37], further strengthening the effect of the HIPK2/HIF-1α balance. Consistently, HIPK2 overexpression reduced the hypoxia-induced angiogenesis in vitro as well as the brain and bone metastasis of the highly metastatic HCC cell line CSQT-2 in a mouse model [37], thus connecting angiogenesis with metastasis [38]. The above reported findings suggest that the lower expression of HIPK2 in cancer tissues, compared to the normal ones, could serve as a novel biomarker of HCC progression due to the HIF-1-induced angiogenesis, although the mechanisms leading to HIPK2 downregulation (e.g., hypoxia or microRNAs) in HCC have not been elucidated and might deserve further studies. The findings also confirm the key role of angiogenesis in the HCC progression and metastasis and highlight how its targeting might represent an efficacious strategy in the clinical treatment of HCC [39]. Antiangiogenic drugs such as sorafenib and regorafenib or the multikinase inhibitors for VEGF receptors, PDGF receptors, and c-Kit, have been shown to be promising therapeutic agents against the HCC, although drug resistance may occur and contribute to the chemotherapeutic failure [40]. Therefore, uncovering novel molecular mechanisms driving angiogenesis in HCC could provide novel potential therapeutical strategies. In this regard, it is tempting to hypothesize that combined therapies including zinc supplementation could, on one hand, inhibit the HIF-1-induced angiogenesis and, on the other hand, restore the HIPK2/p53 antitumor axis, as previously shown [34,35,36].

### 2.2. HIPK2 and microRNA in Tumor Angiogenesis

Another mechanism that underscores the role of HIPK2 in tumor angiogenesis is the microRNA (miRNAs)-induced HIPK2 modulation [41]. miRNAs are non-coding single strand RNAs of about 19–25 nucleotides which bind to the 3′ untranslated region (3′UTR) of target mRNAs to inhibit the translation and induce degradation of the target mRNAs at the post-transcriptional level [42,43]. miRNAs can be included into exosomes, a type of extracellular vesicles that are secreted by many cell types and that contain, other than miRNAs, all the main biomolecules including lipids, proteins, circulating tumor DNA (ctDNA), messenger RNAs, and oncoproteins [44]. Tumor-derived exosomes (TEXs) perform intercellular transfer of components, locally and systemically, interacting with the surrounding cells in the tumor microenvironment. They are considered new players in tumor growth and invasion, tumor-associated angiogenesis, tissue inflammation, and immunologic remodeling [44]. In this regard, it has been found that patients with colorectal cancer (CRC) show high levels of circulating exosomal (exo) miR-1229 which correlated with tumor size, lymphatic metastasis, angiogenesis, and poor survival [45]. Mechanistically, exomiR-1229 targets the HIPK2 3′UTR. Thus, HIPK2 mRNA expression was found to be significantly downregulated in CRC tissues compared to the adjacent normal tissues [45], in agreement with the above-described study showing reduced HIPK2 mRNA expression in CRC tissues compared to the familial adenomatous polyposis (FAP) samples [33]. Circulating exomiR-1229 reduced the HIPK2 protein levels in HUVECs leading to upregulation of the downstream VEGFA, VEGFR1, and p-Akt, thereby stimulating angiogenesis [45]. Hu et al. showed that HIPK2 overexpression counteracts the exomiR-1229-induced upregulation of VEGFA, VEGFR1, and p-Akt, reducing both the extracellular release of VEGF and angiogenesis [45]. The authors identified in the VEGF promoter a potential binding site for myocyte enhancer factor (MEF)-2C, a transcription factor that regulates sprouting angiogenesis directly downstream from VEGFA [46], and demonstrated that the MEF2-mediated activation of VEGF luciferase reporter may be suppressed by HIPK2 [45]. In agreement, a previous study has shown that HIPK2 represses MEF2C-mediated transcriptional activation of VEGF and MMP10, regulating vascular morphogenesis [47]. The authors concluded that the CRC-secreted exomiR-1229 can induce tumor angiogenesis by blocking the HIPK2-mediated suppression of VEGF expression. Hence, lower HIPK2 mRNA or protein levels in CRC tissues compared to the adjacent normal ones can be considered potential novel prognostic biomarkers of CRC progression. In addition, high levels of circulating exomiR-1229, associated with HIPK2 downregulation, could be also considered a potential prognostic biomarker in addition to being a potential therapeutic target for inhibiting tumor angiogenesis in CRC [45].

Interestingly, exomiR-1229 was found to be upregulated in breast cancer and to trigger tumorigenesis by activating the Wnt/β-catenin pathway following targeting the key negative regulators of β-catenin such as glycogen synthase kinase (GSK)-3β, adenomatous polyposis coli (APC), and ICAT [48]. The Wnt/β-catenin pathway is an evolutionarily conserved cellular signaling system involved in different biologic processes such as organogenesis, tissue homeostasis, as well as in the pathogenesis of many human diseases [49]. The β-catenin transcription factor indeed induces the expression of several target genes involved in cell growth and angiogenesis including c-myc, cyclin D1, and VEGF [50]. The β-catenin transcription factor is strongly involved in the early and stepwise events of the colon tumorigenesis and an aberrant activation of the Wnt/β-catenin signaling has been linked to the progression of many other cancer types [51]. Interestingly, HIPK2 has been shown to phosphorylate and degrade β-catenin protein [52], therefore repressing the β-catenin-induced VEGF expression and tumorigenesis [53,54]. Hence, it is tempting to speculate that high levels of exomiR-1229 might induce tumor angiogenesis not only by blocking the HIPK2-mediated suppression of VEGF expression [45] but also by blocking the HIPK2-mediated inhibition of the β-catenin/VEGF pathway, although this latter hypothesis needs to be supported by further studies. Among the tumor-derived exosomes (TEXs), exomiR-1260b has been shown to target HIPK2 in HUVECs and promote angiogenesis, migration, invasion, and chemoresistance of non-small cell lung cancer (NSCLC) cells [55]. Although the authors did not unveil the molecular mechanisms leading to the increased angiogenesis by exomiR-1260b-induced HIPK2 downregulation, they found a relationship between miR-1260b and HIPK2 and its clinical meaning. They found an inverse correlation between miR-1260b and HIPK2 by analyzing 124 paired NSCLC tissues and adjacent noncancerous lung tissues using quantitative Reverse Transcription (qRT)-PCR. The expression levels of HIPK2 transcripts were significantly lower in NSCLC tissues compared to the corresponding noncancerous lung tissues, while miR-1260b expression was higher in NSCLC tissues compared to the noncancerous lung tissues [55]. Interestingly, HIPK2 downregulation and miR-1260b upregulation correlated with the presence of lymph node and distant metastasis, although the molecular mechanisms were not investigated. Further analyses showed that the level of exomiR-1260b was higher in the plasma of patients with NSCLC compared to that of healthy donors. In addition, Kaplan–Meier survival analysis showed that patients with high exomiR-1260b levels had worse overall survival rates than those with low exomiR-1260b levels [55]. These findings suggest an inverse association between miR-1260b and HIPK2 and underline the new role of low HIPK2 levels as a prognostic indicator or predictor of metastasis in NSCLC. In addition, high levels of exomiR-1260b, associated with HIPK2 downregulation, could be considered a potential prognostic biomarker and a therapeutic target to inhibit NSCLC progression. Interestingly, it has been shown that exomiR-1260b promotes cell invasion through the Wnt/β-catenin signaling pathway in lung adenocarcinoma [56]. Therefore, it can be hypothesized that exomiR-1260b-induced HIPK2 downregulation can consequently inhibit also the β-catenin signaling leading to angiogenesis and metastasis, as reported above for the exomiR-1229 [48].

### 2.3. HIPK2 and Circular RNA in Tumor Angiogenesis

miRNAs can be regulated by circular RNAs (circRNAs), a large family of non-coding (nc) RNAs which are produced by “back splicing” of primary transcripts, and are more stable in vivo because they are protected from exonuclease degradation [57,58]. Dysregulation of circRNAs is associated with the development of many diseases; hence, they are considered potentially useful biomarkers [59]. In this regard, high expression of circHIPK2 was found in cisplatin (DDP)-resistant NSCLC cells and tissues [60]. Bioinformatic analyses predicted that miR-1249–3p was the downstream target of circHIPK2, and the authors found that miR-1249–3p was indeed downregulated in NSCLC tissues and cells [60]. The VEGFA expression positively correlated with circHIPK2 while negatively correlating with miR-1249–3p expression, as assessed by tumor xenograft studies. The authors showed that miR-1249–3p is a regulator of VEGFA expression and that VEGF was responsible of induction of angiogenesis and resistance to cisplatin. At the biological level, circHIPK2 silencing in lung cancer A549-DDP-resistant cells reduced their proliferation and inhibited the tube formation of HUVEC, leading to reduced tumor growth in vivo [60]. They concluded that circHIPK2 has the malignant property to induce angiogenesis in NSCLC via miR-1249–3p/VEGF axis [60]. High levels of circHIPK2 are starting to be found in a few other tumors and are being associated with increased tumor progression, although angiogenesis was not always analyzed in those studies [61,62,63]. A high level of circHIPK2 has been found in CRC tissues compared to the adjacent normal tissue, and has been associated with lower overall and disease-free survival rate [64]. CircHIPK2 has been found remarkably upregulated in nasopharyngeal carcinoma (NPC) tissues [65]. In vitro and in vivo studies in animal models showed that circHIPK2 promotes proliferation of NPC cells while knockdown of circHIPK2 dampens the growth of NPC cells [65]. Mechanistically, circHIPK2 downregulated HIPK2 at the protein levels and consequently increased the β-catenin protein expression. Hence, high levels of circHIPK2 have potential clinical significance in CRC and NPC progression; therefore, analysis of circHIPK2 may be worth of further studies also in other tumor types. The biological consequences of miRNAs-induced HIPK2 targeting and of the high circHIPK2 levels in tumors are summarized in Table 1.

## 3. HIPK2 and Other Angiogenic Diseases

### 3.1. HIPK2 and Angiogenesis in Gestational Complications and in Myocardial Infarction (MI)

Gestational hypertension is the second leading cause of maternal death in developed countries [66]. Angiogenesis plays a role in gestational hypertension through upregulation of angiopoietin-1 (ANG-1) or activation of the renin angiotensin system (RAS) that causes high circulating levels of angiotensin-II (ANG-II) [67]. HIPK2 has been shown to play a role in angiogenesis of a model of gestational hypertension induced by hypoxia and reoxygenation (H/R). Human placental microvascular endothelial cells (HPMECs) undergoing H/R showed downregulation of miR-100-5p along with reduced concentrations of ANG-1 and ANG-2 and reduced VEGFA, TGF-β, and PLGF protein levels that correlated with reduced viability and angiogenesis of HPMECs [66]. Rescue assays showed that miR-100-5p overexpression promoted HPMECs viability and angiogenesis restoring the levels of ANG-1, ANG-2, VEGFA, TGF-β, and PLGF inhibited by H/R [68]. Interestingly, miR-100-5p overexpression significantly downregulated the expression levels of HIPK2 in HPMECs and, indeed, HIPK2 was found to targeted and negatively modulated by miR-110-5p [68]. The authors showed that HIPK2 overexpression decreases the expression of VEGFA and TGF-β while increases the expression of anti-angiogenetic proteins (e.g., sFLT1 and sENG). Such overexpression reversed the effect induced by overexpression of miR-100-5p in terms of viability and angiogenesis in HPMECs exposed to the H/R. Mechanistically, miR-100-5p-induced HIPK2 downregulation led to the activation of the PI3K/AKT signaling pathway and such activation was reversed by HIPK2 overexpression [68].

A link between HIPK2 and PI3K/AKT has been previously suggested. In that study, the authors found that the oncogene SPEN induces miR-4652-3p expression in nasopharyngeal carcinoma (NPC) by activation of the PI3K/AKT/c-JUN signaling and that miR-4652-3p targets and downregulates HIPK2 [69]. The PI3K/AKT inhibitor LY294002 counteracted the increase in HPMEC viability and angiogenesis induced by miR-100-5p overexpression, an effect that was further strengthened by HIPK2 overexpression [68]. Given the lack of effective therapies against pregnancy-induced hypertension, the discovery of new potential therapeutic targets such as the miR-100-5p could help to reduce the overall risk of cardiovascular, cerebrovascular, kidney diseases, and diabetes during pregnancy. Another complication that may occur during pregnancy is the increased risk of bronchopulmonary dysplasia (BPD) promoted by smoking [70]. In a mouse model of gestational exposure to sidestream cigarette smoke (SS), the BPD-like condition correlated with impaired angiogenesis, suppression of VEGF, and increase in the alveolar cells’ apoptosis [71]. Mechanistically, gestational SS inhibited HIF-1α and increased pro-apoptotic factors including HIPK2 [71], in line with the role of HIF-1α in inducing the HIPK2 degradation, an interplay that is known to affect both apoptosis and angiogenesis [31].

Among the miRNAs that regulate angiogenesis there is miR-126 that has been shown to directly repress the negative regulators of the VEGF pathway, including the Sprouty-related protein SPRED1 and the phosphoinositol-3 kinase regulatory subunit 2 (PIK3R2) [72]. More recently, miR-126-5p was investigated in a model of myocardial infarction (MI), the most common cardiovascular disease in which hypoxia induces endothelial injury [73]. The authors found that miR-126-5p was upregulated in hypoxia-treated HUVECs undergoing oxidative stress and apoptosis, an effect that was counteracted by inhibiting miR-126-5p via negative regulation of HIPK2 which was predicted as a target of miR-126-5p. However, the molecular mechanisms of miR-126-5p-induced HIPK2 regulation were not investigated in this study, neither was the ability of HUVEC to undergo angiogenesis [74]. The biological consequences of miRNAs-induced HIPK2 targeting in angiogenic diseases are summarized below in Table 2.

### 3.2. HIPK2 in Diabetic Retinopathy (DR) and in Diabetic Wound Healing

Diabetic retinopathy (DR) is the primary cause of blindness in the world. It is a complication of diabetes characterized by hyperglycemia that damages retina [75,76] and has limited treatments options [77]. A key role in the vascular complications of DR has been described for several classes of non-coding RNA including miRNAs [78]. It has been shown that miR-4235-5p can increase proliferation, migration, and angiogenesis of retinal endothelial cells (RECs) cultured in high glucose (HG) condition [79]. In agreement, elevated miR-423-5p levels were found to be present in the plasma of DR patients [79]. RECs cultured in HG showed E2F1-dependent miR-423-5p upregulation that was responsible of HIPK2 downregulation and of HIF-1α and VEGF upregulation [79]. Knockdown of E2F1 or miR-423-5p suppressed the HG-induced angiogenesis and restored the HIPK2 levels [79]. In vivo studies in a mouse model of streptozotocin (STZ)-induced diabetes confirmed that VEGF was upregulated in the retina in correlation with the upregulation of E2F1 and miR-423-5p and the downregulation of HIPK2 [79]. These data suggest that HIPK2 acts as a suppressor of angiogenesis in DR, likely through downregulation of the HIF-1α/VEGF axis, a role played also in angiogenesis during cancer development [31]. The above reported study shed some light into the mechanisms driving DR progression and identified promising biomarkers, such as low HIPK2 levels, and potential targets, such as elevated miR-423-5p levels in the plasma, to predict the disease progression and to eventually design novel therapeutic strategies. The low expression of the HIPK2 levels in DR is also in agreement with our recent study showing that hyperglycemia triggers HIPK2 degradation via HIF-1, increasing tumor progression [80,81,82,83].

Another frequent complication of hyperglycemia is the diabetic foot ulcer [84,85], a consequence of neurological disorders and peripheral vascular complications due to impaired angiogenesis that leads to reduced wound healing and increased risk of infections [86]. It has been previously shown that endothelial progenitor cell-derived exosomes containing miR-221-3p alleviate diabetic ulcers improving wound healing [87]. Subsequently, it has been shown that HG inhibited HUVEC migration and capillary formation, effects that could be reversed by treatment with miR-221-3p that promoted angiogenesis and improved the wound healing [85]. The authors of this study also found an increased expression of HIPK2 in skin tissues of diabetic mice when compared to normal ones, as well as in HG-cultured HUVECs [88]. This is in agreement with a finding showing that HG, by downregulating Siah1, increases HIPK2 expression in glomerular mesangial cells of a mouse model of diabetic nephropathy [89]. HIPK2 inhibition with small interfering (si) RNA rescued HUVEC migration and tube formation under HG condition, while it did not affect HUVEC migration and tube formation in normal metabolic condition [88]. HIPK2 was found to be targeted and negatively regulated by miR-221-3p. Subcutaneous injection of miR-221-3p agomir (which upregulates miRNA activity) into diabetic mice, suppressed HIPK2 expression in wound margin tissues and promoted wound healing [88]. These findings indicate that HG condition reduces angiogenesis and impairs wound healing, effects that correlated with the increased expression of HIPK2. Although the authors did not elucidate the molecular mechanisms through which miR-221-3p/HIPK2 may affect angiogenesis in diabetic condition, they suggest that miR-221-3p analogs may be potentially useful for treating diabetic foot ulcers and for improving wound healing [88]. The biological consequences of miRNAs-induced HIPK2 targeting in angiogenic diseases are summarized in Table 2.

**Table 2:** Summary of the miRNA/HIPK2 expression described in the reported references.

miRNA	Cell Type	Disease Model	Target	Reference
miR-100-5p	HPMEC ^1^	Gestational hypertension	↓ HIPK2	[68]
miR-126-5p	HUVEC ^2^	Myocardial infarction (MI)	↑ HIPK2	[74]
miR-423-5p	REC ^3^	Diabetic retinopathy	↓ HIPK2	[79]
miR-221-3p	HUVEC ^2^	Diabetic foot ulcer	↓ HIPK2	[85]

^1^ HPMEC: human placental microvascular endothelial cell; ^2^ HUVEC: human umbilical vein endothelial cell; ^3^ REC: retinal endothelial cell; ^4^ NPC: nasopharyngeal carcinoma; ↓: downregulation; ↑: upregulation.

## 4. Conclusions

The studies performed in more than twenty years since its discovery have depicted HIPK2 as a central hub in a molecular network that controls several signaling pathways involved in cell death and proliferation and that restrain tumor growth. In this scenario, HIPK2 downregulation by hypoxia-driven mechanisms plays a key role in inducing tumor angiogenesis and solid tumor progression. The role of HIPK2 in restraining tumor angiogenesis has been strengthened by several studies also showing that miRNAs may induce HIPK2 downregulation. Based on these findings, mostly obtained in pre-clinical studies, we can hypothesize that low HIPK2 mRNA or protein levels in cancer tissues compared to the adjacent normal ones can be considered a potential novel prognostic biomarkers of cancer progression, especially if correlated with increased angiogenesis. Interestingly, HIPK2 downregulation by some miRNAs has been shown to be involved in diabetic retinopathy, diabetic wound ulcer, and gestational hypertension, by limiting HIF-1-induced VEGF, and/or β-catenin-induced-VEGF or by activating p53.

Defining the molecular basis of angiogenic disorders in greater detail may provide new avenues to improve the prognosis of angiogenic diseases including cancer and to develop more tailored therapeutic strategies. In this regard, the lower expression of HIPK2 in angiogenic tissues compared to the normal ones could become a novel biomarker of angiogenic diseases that deserves to be supported by further studies.

## Figures and Tables

**Table 1:** Summary of the miRNA/HIPK2 and circHIPK2 expression described in the reported references.

miRNA/circHIPK2	Tumor Type	Biological Effect	Target	Tissues	Cell Lines	Reference
miR-1229	CRC ^1^	angiogenesis, metastasis	↓ HIPK2	+	+	[45]
miR-1260b	NSCLC ^2^	angiogenesis, metastasis	↓ HIPK2	+	+	[55]
circHIPK2	DDP-resistant NSCLC ^3^	angiogenesis, drug resistance	↓ miR-1249-3p, ↑ VEGF	+	+	[60]
circHIPK2	CRC ^1^	reduced overall survival	?	+	+	[64]
circHIPK2	NPC ^4^	tumor progression	?	+	+	[65]

^1^ CRC: colorectal cancer; ^2^ NSCLC: non-small cell lung cancer; ^3^ DDP-resistant: cisplatin resistant; ^4^ NPC: nasopharyngeal carcinoma; ↓: downregulation; ↑: upregulation; +: present in analyzed tissues and cell lines; ?: not determined.
